# Studying the prominence effect amid the COVID-19 crisis: implications for public health policy decision-making.

**DOI:** 10.12688/f1000research.27324.2

**Published:** 2021-05-07

**Authors:** Yossi Maaravi, Ben Heller

**Affiliations:** 1 The Adelson School of Entrepreneurship, Interdisciplinary Center (IDC), Herzliya, Israel, 4610101, Israel; 2 Baruch Ivcher School of Psychology, Interdisciplinary Center (IDC), Herzliya, Israel, 4610101, Israel

**Keywords:** Covid-19, Prominence effect, Decision making, Public policy

## Abstract

The novel coronavirus disease 2019 (COVID-19) has brought with it crucial policy- and decision-making situations, especially when making judgments between financial and health concerns. One particularly relevant decision-making phenomenon is the prominence effect, where decision-makers base their decisions on the most prominent attribute of the object at hand (e.g., health concerns) rather than weigh all the attributes together. This bias diminishes when the decision-making mode inhibits heuristic processes. In this study, we tested the prominence of health vs. financial concerns across two decision-making modes - choice (prone to heuristics) and matching (mitigates heuristics) - during the peak of the COVID-19 in the UK using Tversky *et al.*’s classic experimental paradigm. We added to the classic experimental design a priming condition. Participants were presented with two casualty-minimization programs, differing in lives saved and costs: program X would save 100 lives at the cost of 55-million-pound sterling, whereas program Y would save 30 lives at the cost of 12-million-pound sterling. Half of the participants were required to choose between the programs (choice condition). The other half were not given the cost of program X and were asked to determine what the cost should be to make it as equally attractive as the program Y. Participants in both groups were primed for either: a) financial concerns; b) health concerns; or c) control (no priming). Results showed that in the choice condition, unless primed for financial concerns, health concerns are more prominent. In the matching condition, on the other hand, the prominence of health concerns did not affect decision-makers, as they all “preferred” the cheaper option. These results add further support to the practical relevance of using the proper decision-making modes in times of consequential crises where multiple concerns, interests, and parties are involved.

## Introduction

In just a few months, coronavirus disease 2019 (COVID-19) has spread globally, taking lives and undermining economies ^1^. Consequently, the World Health Organization announced a global emergency, and countries have taken numerous measures to fight it: isolation, quarantine, social distancing, etc. ^2^.

While these measures have clear health advantages, their effect on economies and the financial situation of individuals has been disastrous ^3^: research has shown that social distancing measures, monetary and public health policy decisions, and the shutdown of international travel severely affected economic activity and the stock market ^4^. This tension between health and financial interests has led to numerous debates surrounding one question: how to judge different alternatives (e.g., two different programs to fight the pandemic) when they vary across more than one criterion (e.g., their death toll vs. their financial impact)?

Research in behavioral decision-making has long shown that decision-makers diverge significantly from the rational “ *homo-economicus*” of classic economics ^5^. Researchers suggested that uncertainty is a breeding ground for irrational or biased judgments that are based on emotions and heuristics ^6^. Such patterns persist even during crucial decisions (e.g., health issues ^7^) and those made by professionals (e.g., courtroom judges ^8^).

A relevant behavior is people’s tendency to overly focus on a single dominant attribute of objects they judge ^9^. One particularly apt bias is the *prominence* effect - people’s tendency to focus on the most prominent attribute of judged objects instead of weighing all criteria ^10^. Tversky *et al.* (1988) presented decision-makers a problem where health and economic considerations were mixed, a paradigm aptly suitable for research into the current situation. Using two decision modes (i.e., choice vs. matching), they showed that people tend to overly focus on the more salient attribute of casualties rather than costs. As detailed below, we used the same scenario amid the COVID-19 crisis with the following changes: the virus instead of traffic accidents and a town in the UK instead of Israel. 

We hypothesized that unless decision mode (i.e., matching) or role manipulation deliberately made participants pay more attention to the financial criterion, they would overly emphasize casualties over costs.

## Methods

### Study design

Participants from the UK (129 males and 291 females, all adults above 18 years of age) were recruited through the “ Prolific” [22/4/2020] crowd-working platform and answered an online questionnaire for a pay. The only criterion for participation was that participants be above 18 years of age. Other than this requirement, participants were sampled in a “first come first serve” manner: whoever signed up for the study and was eligible to participate before reaching our target number of participants could answer the questionnaire. The participants were randomly assigned to the different experimental conditions. The study was conducted on the 22 ^nd^ of April, 2020.

### Scenario

Participants read a scenario regarding the predicted number of COVID-19 related casualties (600) over the next year in a small town in the UK. They were then required to consider two programs (X vs. Y), described in terms of yearly costs (in millions of Pound Sterling £) and the number of casualties. Participants were randomly assigned to one of three groups, and were asked to imagine themselves as either: Policymakers in the 1. Ministry of Treasury; 2. Ministry of Health; 3. Control group participants were not given a role.

*Decision measure*. Half of the participants were required to choose between the two programs (choice condition): program X would lower casualties to 500 at the cost of 55-million-pound sterling, and program Y would lower casualties to 570 at the cost of 12-million-pound sterling. The output data for this condition was proportion of participants chosing program X vs. Y (See results section).

The other half received the same information except that program X’s price was missing. They were required to determine a cost that would make it as equally attractive as program Y (matching condition). To do so, they were first asked whether 55-million-pound sterling was the right number and to then type the appropriate cost. Missing casualty information was not used as another condition since Tversky *et al.* (1987) showed that the prominence effect occurred regardless of the dimension left missing. See the extended data sub-section at the end of this article for a complete description of the scenario and questionnaire ^11^.

### Analysis

All statistical analyses were conducted using IBM SPSS Statistics for Windows, Version 22.0. In order to test for differences in proportion of participants who chose each program within each role (health, treasury, control) we conducted a binomial test, and to test for significant differences in these proportions between each role we conducted two chi-squared tests. Additionally, in order to test for differences in proportion of participants who thought that 55 million Pound Sterling was too high/low of a matching price within each role we conducted a binomial test. Finally, three single sample t-tests with 40 million Pound Sterling as a criterion were conducted in order determine whether participants within each role were willing to pay the corrected (triangulated) price.

### Ethical approval

This study was approved by the IRB at the Adelson School of Entrepreneurship, The Interdisciplinary Center, Herzliya (IRB confirmation no. 9). Informed consent was gathered from participants prior to their starting the study. They were given details as to the general topic of the study (decision making), the expected duration (5 minutes), and were assured that their answers were to be anonymously collected and no unsolicited use of their data would occur. Finally, they were informed that they may decide to not participate in the study after reading this information and could stop participating at any time if the study made them feel uncomfortable or for any reason.

## Results and discussion

**Figure 1.**
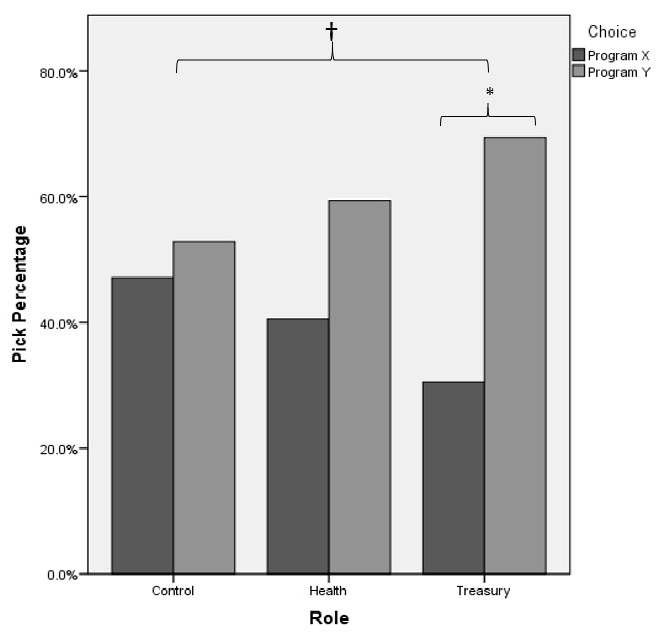
Program Choice Percentage. Figure depicting the percentage of participants who chose each program within each role. *p<0.001, †p<0.05.

*Choice condition.* A binomial test indicated that the proportion of participants in the treasury group who chose program Y (69.4%) over program X (30.6%) was significantly greater than 50% (chance value), p = 0.001 (2-sided; see Figure 1 ^11^). There was no significant difference in these proportions for participants in both the health (Y= 59.4%, X= 40.6%) and the control groups (Y= 52.9%, X= 47.1%). Additionally, Two chi-square tests examined the relationship between role and program-choice proportions. Whereas the proportions of health and control groups did not differ significantly, treasury and control groups were significantly different: χ ^2^ (1, N = 142) = 4.11, p = 0.043 (see Figure 1 ^11^).

*Matching condition.* A binomial test indicated that the proportions of participants in the treasury (95%), health (97%), and control (98%) groups who thought that 55-million-pound sterling was too high a price were significantly greater than 50% (chance value), p < 0.001 (2-sided).

Triangulating lives saved (in comparison to the 600 default) with the cost of program Y, resulted in the appropriate cost of program X being 40-million-pound sterling. Three single sample t-tests with 40 million pounds as the criterion indicated that participants in all roles thought it too high a price: treasury (M= 15.87, SD= 9.44), health (M= 16.43, SD= 7.65), and control (M= 16.19, SD= 9.73) groups: t (65)= -20.74, p< 0.001; t (67)= -25.38, p< 0.001; and t (60)= -19.10, p< 0.001 respectively. The same analysis using 20 as a stricter criterion yielded similar results in the treasury (t (65)= -3.54, p= 0.001), health (t (67)= -3.84, p< 0.001), and control (t (60)= -3.05, p= 0.003) groups.

At first glance, the results of the *choice* condition seem to diverge from *Tversky et al*.’s prominence effect. There, participants – who were not given any roles - had significantly preferred program X (higher costs, fewer casualties), which implied the prominence of saving lives over costs. But, although our results do not show a significant preference for program X in the control group, 50% of participants still chose it – despite its disproportionate cost to the lives-saved ratio. This finding, coinciding with the beginning of the lockdown in the UK (which made the financial issues much more salient and relevant), lends support to the claim that health issues were still the most prominent. To claim that the financial matters were more salient, we would see a significant preference for program Y. Nevertheless, only the treasury condition yielded this result. Coupled with the results of the matching condition below, it seems like an illustration of the prominence of health issues over financial issues in the choice condition.

When *matching*, participants overwhelmingly preferred the more economical program. Indeed, they thought 55-million-pound sterling was too high of a matching price, and their proposed matching price was significantly lower than 40 and even 20. Comparing these results to the *choice* condition patterns suggests that “ *lives saved”* was the more prominent attribute for participants who chose program X (approximately 50%). Only under the treasury condition did we see a difference in choice favoring the more economical program, demonstrating the prominence effect. 

Taken together, our results support Tversky *et al.*’s prominence effect study. They suggest that decision-makers should *match* the options instead of *choosing* between them in such decision-making situations.

Nevertheless, our study is not without its limitations. First, the costs of both policies were high (in the tens of millions of £) and thus may have been unrelatable to the participants. Future studies should explore the consequences of this limitation by presenting choices with financial costs that are more familiar to participants (e.g. 0.1% of GDP). Second, participants did not have any special expertise in policymaking which, when coupled with the previous limitation, could have resulted in non-robust or generalizable results. Further augmenting this limitation was the fact that the roles (conditions) may have been unrelatable. Thus, futures studies should pool participants with prior professional experience in making these types of decisions in order to increase the generalizability of our results.

## Conclusion

Given the highly consequential decisions policymakers must make in times of crisis, it is crucial to understand which decision-making paradigms might lead to better, less biased decisions. The prominence of health over financial issues may lead to irrational differences between choosing versus matching. Thus, changing the decision-making paradigm from simply choosing alternatives to a matching paradigm can aid policymakers to better weigh all attributes.

## Data availability

### Underlying data

Open Science Framework: Covid-19 Prominence (F1000).

https://doi.org/10.17605/OSF.IO/24Z3G ^11^

This project contains the following underlying data:

- Covid-19_ Prominence (OSF).sav (The original dataset)
- Codebook.xlsx (key to understanding variables in dataset)

### Extended data

Open Science Framework: Covid-19 Prominence (F1000).

https://doi.org/10.17605/OSF.IO/24Z3G ^11^

This project contains the following extended data:

- Covid-19_ Prominence - Scenario.docx (The scenario and questionnaires used in the study)

Data are available under the terms of the Creative Commons Zero "No rights reserved" data waiver (CC0 1.0 Public domain dedication).
