# Supplementary material for: Studying the prominence effect amid the COVID-19 crisis: implications for public health policy decision-making
Source: F1000Res. 2021 May 7;9:1356. Originally published 2020 Nov 20. [Version 2] doi: 10.12688/f1000research.27324.2 (PMC8112458; doi:10.12688/f1000research.27324.2)
Supplement: Peer review history

# Peer review: Reviewer response for version 2

**DOI:** 10.5256/f1000research.56449.r84800

**Published:** 2021-05-10

**Authors:** Roshen Fernando

**Affiliations:** 1 Crawford School of Public Policy, Australian National University, Canberra, ACT, Australia

The responses from the authors are satisfactory. Yet, most of the comments have been acknowledged only as limitations of the study, rather than the limitations, including use of figures from a 1987 study, being fixed.

Is the work clearly and accurately presented and does it cite the current literature?

Partly

If applicable, is the statistical analysis and its interpretation appropriate?

Yes

Are all the source data underlying the results available to ensure full reproducibility?

Partly

Is the study design appropriate and is the work technically sound?

No

Are the conclusions drawn adequately supported by the results?

No

Are sufficient details of methods and analysis provided to allow replication by others?

Partly

Reviewer Expertise:

Economic Policy, Statistics, Health Economics

I confirm that I have read this submission and believe that I have an appropriate level of expertise to confirm that it is of an acceptable scientific standard.

# Peer review: Reviewer response for version 1

**DOI:** 10.5256/f1000research.30193.r81287

**Published:** 2021-04-15

**Authors:** Peterson K Ozili

**Affiliations:** 1 Essex Business School, University of Essex, Wivenhoe Park, Colchester, UK

The article examines an important issue about the COVID-19 pandemic – how people choose between casualties (a health consideration) over costs (an economic consideration). The authors assess whether people would choose casualty over cost, and whether they do so when their attention is drawn to more specific economic realities. The method used conform to ethics such as using adult participants, transparent payment for participation, informed consent of participants, equal amount of time for experiment, etc.

 Although the number of females exceed the male participants, I don’t think the skewed gender size matters as it may not affect the result because the reaction of males and females to unpleasant experiences is relatively the same in most cases. The research instrument and design is appropriate, and more interestingly, the findings support the prominence effect of Tversky *et al. *(1988) ^1^ *.*

 In the conclusion section, please suggest some areas for future research. In section 1, no citations were provided to support the claim about the conflcting health tension (e.g. to save people) and the economic tension (to save the economy). The authors can try to expand on why the economic cost matters during the pandemic. To address this, I suggest a few papers that could help the authors in the regard, especially, McKibbin & Fernando (2021) ^2^, and Ozili and Arun (2020) ^3^. These two articles clearly breakdown the economic costs of the pandemic on individuals, firms and countries. The second paper is one of my articles on the economic consequences of COVID-19.

Is the work clearly and accurately presented and does it cite the current literature?

Partly

If applicable, is the statistical analysis and its interpretation appropriate?

Yes

Are all the source data underlying the results available to ensure full reproducibility?

Yes

Is the study design appropriate and is the work technically sound?

Yes

Are the conclusions drawn adequately supported by the results?

Yes

Are sufficient details of methods and analysis provided to allow replication by others?

Yes

Reviewer Expertise:

Economic policy making

I confirm that I have read this submission and believe that I have an appropriate level of expertise to confirm that it is of an acceptable scientific standard.

## References

[1] : Contingent weighting in judgment and choice. *Psychological Review*.1988;95(3) : 10.1037/0033-295X.95.3.371 371-384 10.1037/0033-295X.95.3.371

[2] : The Global Macroeconomic Impacts of COVID-19: Seven Scenarios. *Asian Economic Papers*.2021; 10.1162/asep_a_00796 1-30 10.1162/asep_a_00796

[3] : Spillover of COVID-19: Impact on the Global Economy. *SSRN Electronic Journal*.2020; 10.2139/ssrn.3562570 10.2139/ssrn.3562570

# Response

**Published:** 2021-04-24

**Authors:** Yossi Maaravi

**Affiliations:** The Interdisciplinary Center, Herzliya, Israel

**Reviewer 2**

*The article examines an important issue about the COVID-19 pandemic – how people choose between casualties (a health consideration) over costs (an economic consideration). The authors assess whether people would choose casualty over cost, and whether they do so when their attention is drawn to more specific economic realities. The method used conform to ethics such as using adult participants, transparent payment for participation, informed consent of participants, equal amount of time for experiment, etc.*

**

*Although the number of females exceed the male participants, I don’t think the skewed gender size matters as it may not affect the result because the reaction of males and females to unpleasant experiences is relatively the same in most cases. The research instrument and design is appropriate, and more interestingly, the findings support the prominence effect of Tversky et al. (1988) ^1^.*

**Response:**

 Thank you for taking the time to review our manuscript. We appreciate your comments and suggestions, and we are positive that thanks to them, our manuscript will be better suited for publication.

*In the conclusion section, please suggest some areas for future research.*

**Response:**

 Thank you for this suggestion. We also believe that this study offers multiple opportunities for future research endeavors. Therefore, we have added a paragraph discussing the limitations of our study and possible future research directions.

*In section 1, no citations were provided to support the claim about the conflcting health tension (e.g. to save people) and the economic tension (to save the economy). The authors can try to expand on why the economic cost matters during the pandemic. To address this, I suggest a few papers that could help the authors in the regard, especially, McKibbin & Fernando (2021)* *^2^* *, and Ozili and Arun (2020) ^3^. These two articles clearly breakdown the economic costs of the pandemic on individuals, firms and countries. The second paper is one of my articles on the economic consequences of COVID-19.*

**Response:**

 Thank you for this remark and your recommendations. We agree that this point could have been made in a more comprehensive fashion, and have thus elaborated on the health-economy tradeoff. Your suggested papers have proven to be very informative and useful in this regard.

# Peer review: Reviewer response for version 1

**DOI:** 10.5256/f1000research.30193.r79762

**Published:** 2021-03-17

**Authors:** Roshen Fernando

**Affiliations:** 1 Crawford School of Public Policy, Australian National University, Canberra, ACT, Australia

The brief report titled, assesses the implications of the prominence effect among the health and economic concerns in decision making in the public health sphere during a pandemic. The study evaluates the impact of two decision modes: choice vs matching. The authors use the classic experimental paradigm to base the survey and uses few statistical techniques to analyze the findings. The study concludes the importance of matching decision making mode in times of a pandemic.

 The paper is well-written though quite brief. It is important to rethink whether the brief report could replace a more descriptive journal article format, especially when communicating finer points of the study. Otherwise, the value contribution to the body of knowledge could be minimal.

 The study suffers primarily from two major limitations. Firstly, the authors seem to have confused the difference between financial and economic outcomes. What the authors involve in the study are financial outcomes, which are not in any way a substitute to the economic outcomes. In fact, healthy populations are central to an economy and during a pandemic, it is essential to restore confidence in the economy by controlling the infection spread as soon as possible. One lesson COVID-19 demonstrates is that the economic outcomes of countries which did not adopt lockdowns are not different to those who adopted lockdowns. These observations make the health vs economy a false debate. Unless the authors want to get into this debate, which clearly cannot be done using a brief report, the mentioning of economic outcomes should be replaced with financial outcomes, and the research should be framed accordingly.

 Secondly, the roles assigned to the participants, who are clearly submitting to the study due to a financial incentive, is not robust. I believe that the authors could figure out an alternative way to address the process of assigning roles. The financial figures presented also are arbitrary and it is questionable what the figures could have meant for the participants. Using something relatable, say 0.1% of GDP, could have made the process more robust.

 I suggest that the authors review their methodology and improve the results for it to be impactful.

Is the work clearly and accurately presented and does it cite the current literature?

Partly

If applicable, is the statistical analysis and its interpretation appropriate?

Yes

Are all the source data underlying the results available to ensure full reproducibility?

Partly

Is the study design appropriate and is the work technically sound?

No

Are the conclusions drawn adequately supported by the results?

No

Are sufficient details of methods and analysis provided to allow replication by others?

Partly

Reviewer Expertise:

Economic Policy, Statistics, Health Economics

I confirm that I have read this submission and believe that I have an appropriate level of expertise to state that I do not consider it to be of an acceptable scientific standard, for reasons outlined above.

# Response

**Published:** 2021-04-24

**Authors:** Yossi Maaravi

**Affiliations:** The Interdisciplinary Center, Herzliya, Israel

**Reviewer 1**

*The brief report titled, assesses the implications of the prominence effect among the health and economic concerns in decision making in the public health sphere during a pandemic. The study evaluates the impact of two decision modes: choice vs matching. The authors use the classic experimental paradigm to base the survey and uses few statistical techniques to analyze the findings. The study concludes the importance of matching decision making mode in times of a pandemic.*

**

*The paper is well-written though quite brief. It is important to rethink whether the brief report could replace a more descriptive journal article format, especially when communicating finer points of the study. Otherwise, the value contribution to the body of knowledge could be minimal.*

**Response:**

 Thank you for taking the time to review our manuscript. We appreciate your comments and suggestions, and we are positive that thanks to them, our manuscript will be better suited for publication.

*The study suffers primarily from two major limitations. Firstly, the authors seem to have confused the difference between financial and economic outcomes. What the authors involve in the study are financial outcomes, which are not in any way a substitute to the economic outcomes. In fact, healthy populations are central to an economy and during a pandemic, it is essential to restore confidence in the economy by controlling the infection spread as soon as possible. One lesson COVID-19 demonstrates is that the economic outcomes of countries which did not adopt lockdowns are not different to those who adopted lockdowns. These observations make the health vs economy a false debate. Unless the authors want to get into this debate, which clearly cannot be done using a brief report, the mentioning of economic outcomes should be replaced with financial outcomes, and the research should be framed accordingly.*

**Response:**

 Thank you for this important feedback. We have come to agree with your claim that framing the manuscript as a “financial vs. health” trade-off would be more accurate given the relationship between economic prosperity and the population's health in question. We also agree that delving into the differences between financial and economic factors is beyond the scope of our brief report. We have thus modified our manuscript to reflect differences in decision-making modes regarding financial vs. health concerns.

*Secondly, the roles assigned to the participants, who are clearly submitting to the study due to a financial incentive, is not robust. I believe that the authors could figure out an alternative way to address the process of assigning roles.*

**Response:**

 We agree with this contention, yet we believe that the assignment to roles affected participants’ choice - as evidenced by our results - thus allowing us to derive insights from our study. Nevertheless, we have incorporated your feedback as a possible limitation in our manuscript.

*The financial figures presented also are arbitrary and it is questionable what the figures could have meant for the participants. Using something relatable, say 0.1% of GDP, could have made the process more robust.*

**Response:**

 While we agree that these figures may not be relatable and may not mean much to the participants, we used the same figures that Tversky, Sattath, and Slovic used in their seminal article “Contingent Weighting in Judgement and Choice” (1987). We wished to conform as much as possible to their original paradigm while only changing what was necessary to answer our research questions. Nevertheless, we have incorporated your feedback as a limitation in our manuscript.
